# Phase 1 dose-escalation study of a novel oral PI3K/mTOR dual inhibitor, LY3023414, in patients with cancer

**DOI:** 10.1007/s10637-020-00968-5

**Published:** 2020-06-23

**Authors:** Shunsuke Kondo, Masaomi Tajimi, Tomohiko Funai, Koichi Inoue, Hiroya Asou, Vinay Kumar Ranka, Volker Wacheck, Toshihiko Doi

**Affiliations:** 1 Department of Experimental Therapeutics, National Cancer Center Hospital, Tokyo, Japan; 2 Eli Lilly Japan K.K., Kobe, Japan; 3 Eli Lilly Services India Private Limited, Bengaluru, India; 4 Eli Lilly GmbH, Vienna, Austria; 5 Department of Gastrointestinal Oncology, National Cancer Center Hospital East, Chiba, Japan

**Keywords:** LY3023414, Advanced malignancies, Dose-escalation study, Japanese patients, Safety

## Abstract

LY3023414 is an oral, selective adenosine triphosphate-competitive inhibitor of class I phosphatidylinositol 3-kinase isoforms, mammalian target of rapamycin, and DNA-protein kinase in clinical development. We report results of a 3 + 3 dose-escalation Phase 1 study for twice-daily (BID) dosing of LY3023414 monotherapy in Japanese patients with advanced malignancies. The primary objective was to evaluate tolerability and safety of LY3023414. Secondary objectives were to evaluate pharmacokinetics and to explore antitumor activity. A total of 12 patients were enrolled and received 150 mg (*n* = 3) or 200 mg (*n* = 9) LY3023414 BID. Dose-limiting toxicities were only reported at 200 mg LY3023414 for 2 patients with Grade 3 stomatitis. Common treatment-related adverse events (AEs) across both the dose levels included stomatitis (75.0%), nausea (66.7%), decreased appetite (58.3%), diarrhea, and decreased platelet count (41.7%), and they were mostly mild or moderate in severity. Related AEs Grade ≥ 3 reported for ≥1 patient included anemia, stomatitis, hypophosphatemia, and hyperglycemia (*n* = 2, 16.7%). Two patients discontinued due to AEs (interstitial lung disease and stomatitis). No fatal events were reported. The pharmacokinetic profile of LY3023414 was characterized by rapid absorption and elimination. Five patients had a best overall response of stable disease (150 mg, *n* = 3; 200 mg, *n* = 2) for a 55.6% disease control rate. LY3023414 up to 200 mg BID is tolerable and safe in Japanese patients with advanced malignancies.

## Introduction

The phosphatidylinositol 3-kinase (PI3K)/mammalian target of rapamycin (mTOR) pathway has been studied extensively based on its relevance for progression and survival of tumor cells [1–4]. PI3K/mTOR signaling pathway is one of the most frequently activated pathways with alterations in over 70% of human carcinomas including hematologic malignancies and solid tumors such as breast cancer, non-small-cell lung cancer, and endometrial cancer [5–9]. An activated aberrant PI3K/protein kinase B (AKT)/mTOR signaling pathway results in a more aggressive tumor phenotype with enhanced angiogenesis, proliferation, metastases, and drug resistance [10, 11]. A complex feedback loop within the PI3K/AKT/mTOR signaling pathway leads to an activation of compensatory signaling pathways upon individual inhibition of either mTOR or PI3K [12]. To overcome this challenge, it has been suggested that targeting both PI3K and mTOR simultaneously might be an efficient way to avoid activation of the compensatory pathway [6, 7, 10]. Monotherapy with PI3K/mTOR dual inhibitors is of interest in tumor types with a particularly high incidence of aberrant PI3K pathway activation by PIK3CA mutations, amplification, or loss of PTEN tumor suppressor protein [13]. Various dual inhibitors are under investigation for their efficacy and safety [14].

LY3023414 is a novel, oral, selective small-molecule inhibitor of class I PI3K isoforms, mTORC1/2, and DNA-protein kinase with good solubility across a wide pH range [12]. In vitro, LY3023414 showed dose-dependent inhibition of phosphorylation of the PI3K/AKT/mTOR downstream pathway [15]. LY3023414 has shown potent antitumor activity in tumor xenograft models. In a first-in-human Phase 1 study in a Western population comprising patients with cancer, LY3023414 was safe and tolerable up to 200 mg twice daily (BID) as a monotherapy (global recommended dose) and demonstrated single-agent clinical activity in patients with advanced or metastatic cancer. LY3023414 pharmacokinetics (PK) demonstrated dose-dependent increase in exposure with ≥90% target inhibition at doses ≥150 mg. Drug-drug interaction analysis identified LY3023414 as a weak inhibitor of the metabolic clearance of drugs metabolized through CYP3A4 [12].

The aim of this study was to evaluate the tolerability, safety, and PK and to explore preliminary antitumor activity of LY3023414 in Japanese patients with advanced malignancies.

## Material and methods

### Study design

This was a non-randomized, single-arm, open-label, Phase 1, dose-escalation study of oral LY3023414 in Japanese patients with advanced malignancies. A modified 3 + 3 dose-escalation method was used to evaluate the dose-limiting toxicity (DLT) of LY3023414. Enrollment of 3 patients and 6 patients were planned for dose level 1 (150 mg) and dose level 2 (200 mg), respectively. Dose-escalation decisions were made by agreement between investigators and the sponsor, with a consultation with the Safety Assessment Committee (SAC), as needed. If 2 patients experienced DLTs at any given dose, the sponsor examined safety data and consulted the SAC. Following consultation, the sponsor and investigators decided whether the dose was intolerable or additional patients were to be enrolled to the same or an intermediate dose level for further investigation. If ≥3 patients experienced DLTs at any given dose, the dose was considered intolerable for Japanese patients. No intrapatient dose escalation was allowed.

The primary objective of this study was to evaluate the tolerability and safety of LY3023414 up to the global recommended dose in Japanese patients with advanced malignancies. The secondary objectives were to evaluate PK and to document antitumor activity of LY3023414. The exploratory objective was to evaluate pharmacodynamic (PD) effects of LY3023414 on PI3K/mTOR kinase activity.

The protocol was approved by Institutional Review Boards prior to patient recruitment, and each patient provided written informed consent before enrollment. The study was conducted in accordance with consensus ethics principles derived from international ethics guidelines, including the Declaration of Helsinki, the Council for International Organizations of Medical Sciences International Ethical Guideline, and the International Conference on Harmonization E6 Guidelines for Good Clinical Practice.

### Patient population

Japanese patients ≥20 years of age with advanced malignancies who experienced treatment failure with standard therapies, with measurable or non-measurable disease as defined by RECIST Version 1.1, with Eastern Cooperative Oncology Group performance status (ECOG PS) ≤1, and with discontinuation of all previous cancer therapies were included in this study. Exclusion criteria included serious preexisting medical conditions, symptomatic central nervous system malignancies or metastasis, acute or chronic leukemia or current hematologic malignancies, active infections, a secondary primary malignancy, or intolerance to any PI3K/AKT/mTOR inhibitors.

### Study treatment

The study evaluated 2 dose levels, 150 mg or 200 mg LY3023414 monotherapy administered in the morning and evening, approximately 12 h apart. The cycle was of 21 days. Following a 3 + 3 dose-escalation design, the 200-mg BID dose level was administered only after the 150-mg BID dose level was shown to be tolerable. No dose reductions were allowed during Cycle 1, unless a DLT was reported. Dose reductions were allowed in Cycle 2 and beyond at the discretion of the investigator. Patients were treated until a discontinuation criterion was met.

### Safety

Tolerability and safety were assessed through clinical and laboratory evaluations at weekly intervals for the first 2 cycles and at least every 2 weeks thereafter. Adverse events (AEs) were graded according to the Common Terminology Criteria for Adverse Events Version 4.0 and coded to Medical Dictionary for Regulatory Activities preferred terms. All patients who received ≥1 dose of the study drug were included in the safety analysis.

A DLT was defined as any AE during Cycle 1 which was possibly related to study treatment with LY3023414 and fulfilled any of the following main criteria: Grade ≥ 3 non-hematological toxicity (except nausea, vomiting, diarrhea, constipation, anorexia, skin rash, or asymptomatic electrolyte abnormality for ≤3 days and responsive to appropriate treatment; transient ≤5 days hyperglycemia; and transient ≤7 days mucositis), Grade 4 hematological toxicity of ≥7 days, Grade 3 thrombocytopenia with Grade ≥ 2 bleeding or febrile neutropenia, requirement of platelet or packed red blood transfusion, and any toxicities requiring dose omissions of >20% of intended doses in Cycle 1. The maximum tolerated dose was defined as the highest dose of LY3023414 not causing a DLT in more than 33% of patients.

### Pharmacokinetics and pharmacodynamics

For PK assessment, blood samples were collected at pre dose and 0.5, 1, 2, 4, 8, and 12 h post dose on 2 occasions: (i) on Day 1 of Cycle 1 (single dose), and (ii) on Day 15 of Cycle 1 (multiple dose). For PD assessment, blood samples were collected at pre dose and 0.5, 1, 2, and 4 h post dose on 2 occasions: (i) on Day 1 of Cycle 1 (single dose), and (ii) on Day 15 of Cycle 1 (multiple dose).

Blood concentrations of LY3023414 were assayed using a validated dried blood spot liquid chromatography-mass spectrometry/mass spectrometry method at a designated laboratory [16, 17]. The PK parameter estimates for LY3023414 were calculated by standard non-compartmental methods of analysis using Phoenix WinNonlin® 8.0 (Certara, L.P.; Princeton, NJ, USA). The primary parameters for analysis were maximum observed blood concentration (C_max_), time of C_max_ (t_max_), area under the blood concentration-time curve from time zero to the last measurable blood concentration (AUC0-t_last_), and area under the blood concentration-time curve from time zero to infinity (AUC_0-∞_) of LY3023414. Other non-compartmental parameters, such as half-life (t_1/2_), apparent total body clearance (CL/F) on Cycle 1 Day 1, apparent total body clearance at steady state (CLss/F) on Cycle 1 Day 15, and apparent volume of distribution during the terminal phase (Vz/F), were also assessed.

Pharmacodynamic parameters (glucose and C-peptide) were analyzed for all patients. The mean percentage change from baseline against nominal time was provided for glucose and C-peptide.

### Antitumor activity

Tumor response was assessed by computed tomography scans or magnetic resonance imaging according to RECIST v1.1 [18] at baseline and thereafter every 6 weeks until patients discontinued from the study. The assessment included all patients who received ≥1 dose of study drug.

## Results

### Patient disposition and demographics

A total of 16 patients were screened, and 12 patients were enrolled in the study. All patients were Japanese with a median age of 63.5 years (range, 41–73 years) and ECOG PS of 0 (*n* = 10, 83.3%) and 1 (*n* = 2, 16.7%). Half of the patients were male. Most patients (58.3%) had Stage IV disease. Demographics and baseline characteristics are presented in Table 1 for this Phase 1 study population.

**Table 1:** Demographic and baseline characteristics

Parameter	LY3023414	LY3023414	Total
	150 mg	200 mg	(*N* = 12)
	(*n* = 3)	(*n* = 9)	
Age, years, median (range)	64.0 (57.0–73.0)	63.0 (41.0–73.0)	63.5 (41.0–73.0)
< 65 years, n (%)	2 (66.7)	6 (66.7)	8 (66.7)
≥ 65 years, n (%)	1 (33.3)	3 (33.3)	4 (33.3)
Sex, n (%)			
Male	0	6 (66.7)	6 (50.0)
Female	3 (100.0)	3 (33.3)	6 (50.0)
Weight (kg), median (range)	58.2 (53.7–68.3)	56.7 (47.5–87.4)	57.4 (47.5–87.4)
Height (cm), median (range)	154.6 (154.3–156.4)	167.8 (145.5–177.6)	164.9 (145.5–177.6)
BSA, median (range)	1.6 (1.5–1.7)	1.7 (1.5–2.0)	1.6 (1.5–2.0)
ECOG PS, n (%)			
0	3 (100)	7 (77.8)	10 (83.3)
1	0	2 (22.2)	2 (16.7)
Disease at diagnosis, n (%)			
Dedifferentiated liposarcoma	1 (33.3)	0	1 (8.3)
Gallbladder adenocarcinoma	1 (33.3)	0	1 (8.3)
Gastric cancer	0	1 (11.1)	1 (8.3)
Gastrointestinal stromal tumor	0	1 (11.1)	1 (8.3)
Hepatocellular carcinoma	0	1 (11.1)	1 (8.3)
Lung adenocarcinoma	1 (33.3)	1 (11.1)	2 (16.7)
Neoplasm malignant	0	1 (11.1)	1 (8.3)
Pancreatic carcinoma	0	1 (11.1)	1 (8.3)
Rectal adenocarcinoma	0	1 (11.1)	1 (8.3)
Squamous cell carcinoma	0	1 (11.1)	1 (8.3)
Transitional cell carcinoma	0	1 (11.1)	1 (8.3)
Disease stage, n (%)			
Stage III	1 (33.3)	0	1 (8.3)
Stage IIIc	0	1 (11.1)	1 (8.3)
Stage IV	2 (66.7)	5 (55.6)	7 (58.3)
Stage IVa	0	2 (22.2)	2 (16.7)
Unknown	0	1 (11.1)	1 (8.3)
Histopathological diagnosis, n (%)			
Well differentiated	0	3 (33.3)	3 (25.0)
Moderately differentiated	0	1 (11.1)	1 (8.3)
Poorly differentiated	1 (33.3)	0	1 (8.3)
Unable to determine	2 (66.7)	5 (55.6)	7 (58.3)

*BSA* body surface area, *ECOG PS* Eastern Cooperative Oncology Group performance status, *N* number of patients in safety population, *n* number of patients in the specified category

Three patients were enrolled at the 150-mg BID dose level and 9 patients at the 200-mg BID dose level. The median duration of treatment was 13.7 weeks (range, 10.9–16.1) and 6 weeks (range, 0.6–36.1), respectively. The median cumulative dose per patient was 22,500 mg (20,000–33,900 mg) at the 150-mg dose level and 14,700 mg (1600–41,500 mg) at the 200-mg dose level.

### Safety

All 12 patients enrolled in the study were included in the safety analysis. No DLTs were observed for the 3 patients enrolled at the 150-mg LY3023414 dose level. As specified in the study design, 6 patients were enrolled in the 200-mg group. A DLT was observed in 2 out of 6 patients, following which safety monitoring committee recommended to add another 3 patients in the study, for whom, no DLTs were observed. Two out of 9 patients receiving 200 mg LY3023414 BID experienced DLTs of Grade 3 stomatitis. Both DLTs were considered as non-serious by the investigators. In both the patients, the onset of stomatitis was at Day 4. In 1 patient, study treatment was paused on Day 8 through Day 21. The patient did not restart the treatment on Day 22 and discontinued from the study treatment. The DLT resolved on Study Day 36. In another patient, the study drug was omitted at Day 5. The patient did not restart the study drug and withdrew from the study. Therefore, a dose of 200 mg LY3023414 was determined as the maximum tolerated dose for LY3023414 BID dosing.

All patients experienced at least 1 AE considered possibly related to the study drug (Table 2). The most common treatment-related AEs reported in ≥15% of the patients across both dose levels included stomatitis (75.0%), nausea (66.7%), decreased appetite (58.3%), diarrhea, decreased platelet count, anemia (41.7%, each), increased blood creatinine, fatigue, hyperglycemia, hypophosphatemia, proteinuria, vomiting (33.3%, each), increased alanine aminotransferase, increased aspartate aminotransferase, rash, pruritus (25.0%, each), hypokalemia, hypotension, pyrexia, decreased weight, and decreased white blood cell count (16.7%, each).

**Table 2:** Summary of adverse events considered possibly related to study drug (in ≥2 patients in overall population)

Preferred term	Overall AEs (*N* = 12)	150 mg LY3023414 (*n* = 3)		200 mg LY3023414 (*n* = 9)	
	Any grade	n (%)		n (%)	
	n (%)	Any grade	Grade ≥ 3	Any grade	Grade ≥ 3
Stomatitis	9 (75.0)	2 (66.7)	0	7 (77.8)	2 (22.2)
Nausea	8 (66.7)	2 (66.7)	0	6 (66.7)	0
Appetite decreased	7 (58.3)	1 (33.3)	0	6 (66.7)	0
Diarrhea	5 (41.7)	1 (33.3)	0	4 (44.4)	0
Anemia	5 (41.7)	2 (66.7)	1 (33.3)	3 (33.3)	1 (11.1)
Fatigue	4 (33.3)	2 (66.7)	0	2 (22.2)	0
Hyperglycemia	4 (33.3)	2 (66.7)	1 (33.3)	2 (22.2)	1 (11.1)
Hypophosphatemia	4 (33.3)	1 (33.3)	0	3 (33.3)	2 (22.2)
Proteinuria	4 (33.3)	1 (33.3)	0	3 (33.3)	0
Vomiting	4 (33.3)	1 (33.3)	0	3 (33.3)	0
Blood creatinine increased	4 (33.3)	1 (33.3)	0	3 (33.3)	0
ALT increased	3 (25.0)	1 (33.3)	0	2 (22.2)	0
AST increased	3 (25.0)	0	0	3 (33.3)	0
Rash	3 (25.0)	1 (33.3)	0	2 (22.2)	0
Pruritis	3 (25.0)	1 (33.3)	0	2 (22.2)	0
Hypokalemia	2 (16.7)	1 (33.3)	0	1 (11.1)	0
Hypotension	2 (16.7)	0	0	2 (22.2)	0
Pyrexia	2 (16.7)	0	0	2 (22.2)	0
Weight decreased	2 (16.7)	1 (33.3)	0	1 (11.1)	0
Hematological AEs					
Platelet count decreased	5 (41.7)	2 (66.7)	0	3 (33.3)	0
WBC count decreased	2 (16.7)	0	0	2 (22.2)	0

*AEs* adverse events, *ALT* alanine aminotransferase, *AST* aspartate aminotransferase, *N* number of patients in safety population, *n* number of patients in the specified category, *WBC* white blood cell

Two out of 3 patients who received 150 mg LY3023414 experienced drug-related Grade ≥ 3 AEs of anemia and hyperglycemia (33.3%, *n* = 1 each). At the 200-mg LY3023414 BID dose level, 5 out of 9 patients had Grade 3 drug-related AEs, including stomatitis (22.2%, *n* = 2), hypophosphatemia (22.2%, *n* = 2), anemia, decreased lymphocyte count, and hyperglycemia (11.1%, *n* = 1 each). No drug-related Grade 4 or 5 AEs were reported. One Grade 4 event of upper gastrointestinal hemorrhage was reported in a patient with a pancreatic carcinoma tumor; however, it was not considered related to study treatment.

Patients experiencing stomatitis required supportive medication (dexamethasone, azulene, glycerol, lidocaine, morphine hydrochloride, flurbiprofen axetil) and dose omissions for recovery. Patients experienced hyperglycemia, recovered with hyperglycemic medication (insulin, metformin, glimepiride), and did not require study drug dose modification.

Overall, 3 patients experienced serious AEs across both the dose levels, including 2 patients receiving 150 mg LY3023414 (Grade 2 stomatitis and Grade 3 bile duct stenosis) and 1 patient receiving 200 mg LY3023414 (Grade 2 interstitial lung disease [ILD] adjudicated as related to study treatment). The onset of ILD was at Day 51, and the computer tomographic image showed a faint ground-glass opacity pattern. The patient recovered following medical management of the event with intravenous prednisolone and discontinuation of the study treatment.

There were no fatal events due to AEs or any other reason. Two patients receiving LY3023414 200 mg discontinued the study due to AEs of Grade 2 ILD and Grade 1 stomatitis, respectively. Dose adjustments and omissions were required periodically due to AEs (in most patients). Adverse events leading to dose adjustments and omissions included stomatitis (150 mg, *n* = 1; 200 mg, *n* = 2), hypophosphatemia, and abnormal liver function test (200 mg, *n* = 1 each). At the 150-mg dose level, 1 patient (33.3%) had at least 1 dose adjustment, and 2 patients (66.7%) had at least 1 dose omission. At the 200-mg dose level, 3 patients (33.3%) had at least 1 dose adjustment, and all 9 patients had at least 1 dose omission.

### Pharmacokinetics

**Fig. 1:**
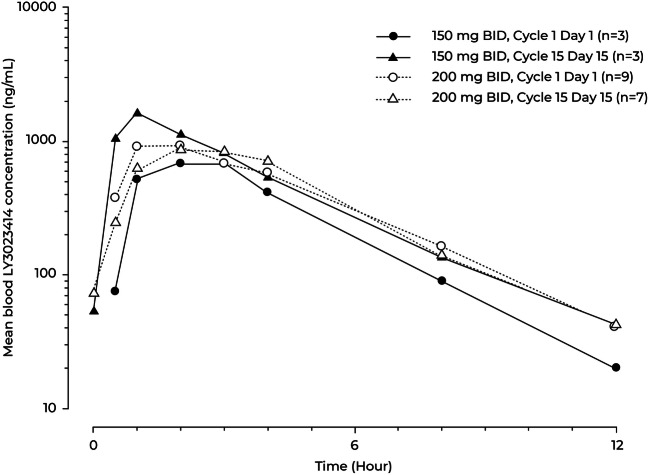
Arithmetic mean blood concentration-time profiles of LY3023414 on Cycle 1 Day 1 and Day 15 following twice-daily (BID) oral doses of 150 mg or 200 mg LY3023414. Note: *N* = 2 for 0.5 h on Cycle 1 Day 1 following LY3023414 150 mg. n; number of pharmacokinetic observations

All 12 patients were included in the PK analyses. The PK profile of LY3023414 showed rapid absorption, with a median t_max_ of 1 to 3 h post administration in each dose level. On study Days 1 and 15 in Cycle 1, the geometric mean t_1/2_ was approximately 2 h for each dose level (Table 3). The PK profiles of LY3023414 were comparable between the 2 dose levels. Blood LY3023414 concentration-time profile is illustrated in Fig. 1.

**Table 3:** Summary of LY3023414 pharmacokinetic parameters

Geometric mean (% CV)				
	150 mg LY3023414		200 mg LY3023414	
	Cycle 1 Day 1	Cycle 1 Day 15	Cycle 1 Day 1	Cycle 1 Day 15
N	3	3	9	7
C_max_ (ng/mL)	792 (22)	1570 (30)	1020 (31)	1010 (39)
t_max_ (hr)^a^	2.93 (0.75–3.03)	1.00 (0.95–1.00)	1.95 (0.95–3.88)	1.97 (0.97–2.95)
t_1/2_ (hr)^b^	1.59 (1.12–1.99)	1.81 (1.48–2.17)	1.94^c^ (1.52–3.15)	1.83 (1.47–2.64)
AUC0-t_last_ (ng hr./mL)^d^	3000 (18)	4920 (44)	4140 (28)	4100 (33)
AUC_0-∞_ (ng hr./mL)	3050 (19)	5010 (46)	4120^c^ (29)	4210 (34)
CL/F (L/hr)^e^	49.2 (19)	30.4 (45)	48.5^c^ (29)	48.6 (33)
V_z_/F (L)	113 (32)	79.3 (27)	136^c^ (35)	129 (32)

AUC, area under the drug plasma concentration versus time curve; AUC_0-∞_, AUC time curve from time zero to infinity; AUC0-t_last_, AUC time curve from time zero to time t_last_, where t_last_ is the last time point with a measurable concentration; CL/F, apparent total body clearance; CL_ss_/F, apparent total body clearance at steady state; C_max_, maximum observed blood concentration; CV, coefficient of variance; N, number of pharmacokinetic observations; PK, pharmacokinetic(s); t_1/2_, half-life associated with the terminal rate constant (lambda z) in non-compartmental analysis; t_max_, time of maximum observed blood concentration; V_z_/F, apparent volume of distribution during the terminal phase

^a^Median (range). ^b^Geometric mean (range). ^c^N = 8. ^d^The last time point in the profile was collected at 12 h post dose (the dosing interval is 12 h); hence, this AUC corresponds to AUCτ (over the dosing interval). ^e^CL_ss_/F was reported on Cycle 1 Day 15 (multiple-dose PK profile), and CL/F was based on AUC_0-∞_ for Cycle 1 Day 1 (single-dose PK profile)

### Pharmacodynamics

**Fig. 2:**
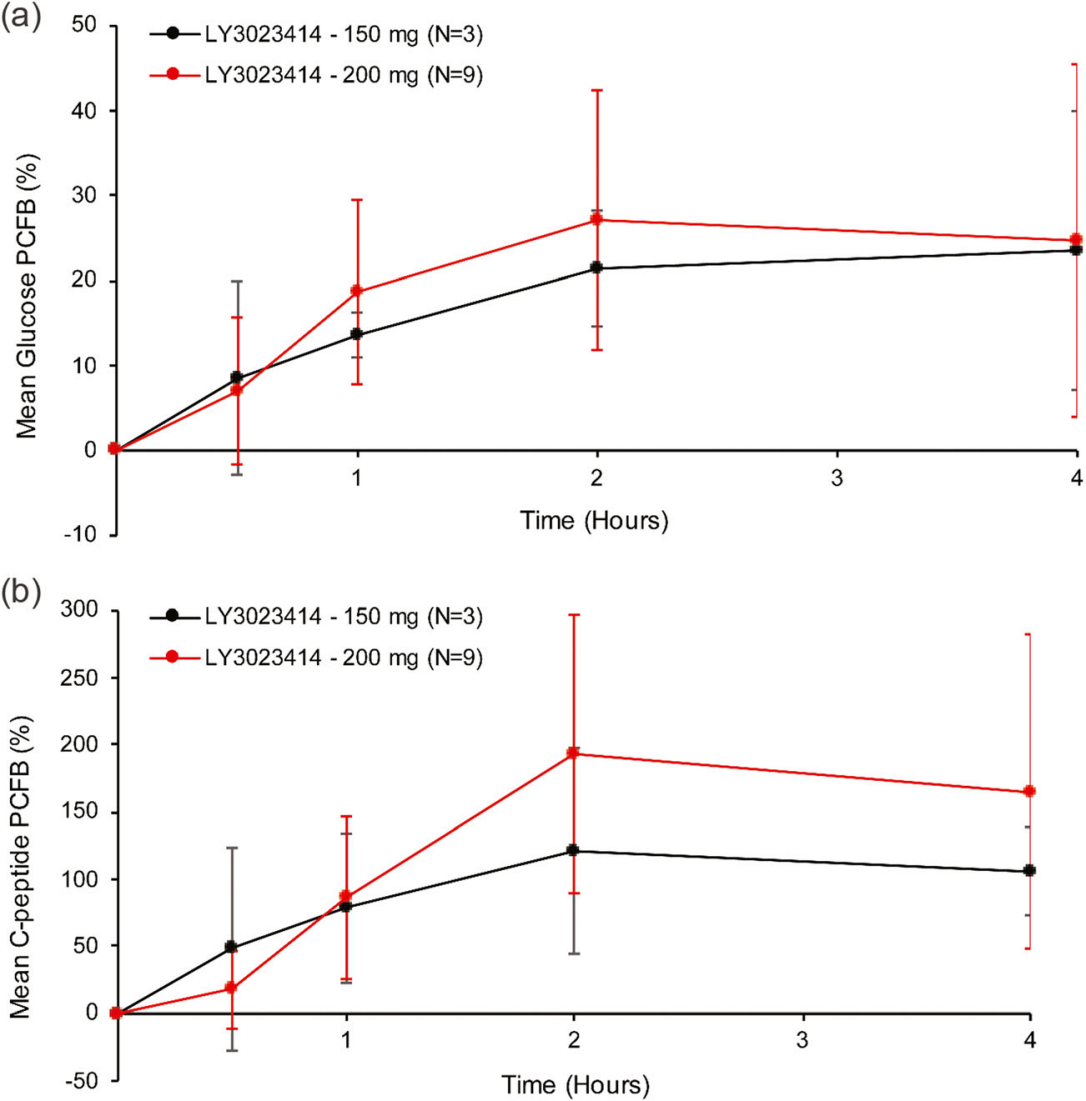
**a** Mean glucose percentage change and **b** mean C-peptide percentage change from baseline (PCFB) after single-dose (Cycle 1 Day 1) administration of 150 mg or 200 mg LY3023414. Note: Oral administration of LY3023414 occurred at time 0 on fasted/empty stomach, and patients continued to fast up to 4 h post dose. A meal could be given after 4 h post dose. I bars indicate standard deviation. N; number of patients in the specified category

Increased levels of fasting glucose and C-peptide were observed after LY3023414 (150 or 200 mg) administration (Fig. 2). The mean percentage change from baseline to 4 h post dose in glucose and C-peptide were 23.6% and 106.0% for patients on 150 mg and 24.7% and 165.4% for patients on 200 mg, respectively. Considering the high variability in the measurements of PD markers at each time point, their mean levels appear overall comparable between the 2 doses (Fig. 2). This transient increase in glucose and C-peptide is consistent with previous studies for PI3K/mTOR inhibitors and expected as a PD effect based on the biology and the mechanism of action of this class of drugs.

### Antitumor activity

**Fig. 3:**
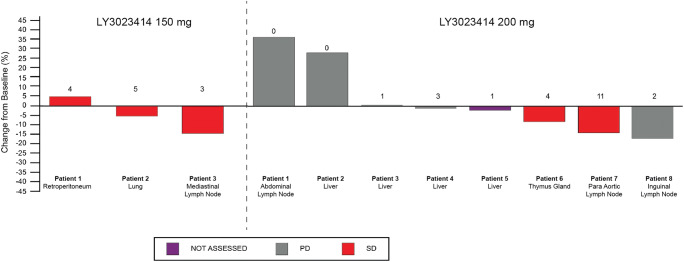
Waterfall plot of best percentage change in summation of largest target lesion for patients receiving 150 mg and 200 mg LY3023414. Numbers on the bars represent number of completed dosing cycles by patient. PD, progressive disease; SD, stable disease

Of 12 patients, 9 patients had at least 1 postbaseline tumor scan for response assessment and were considered evaluable according to RECIST v1.1. No complete response (CR) or partial response (PR) was reported. Summation of patients’ target lesions has been shown in Fig. 3. A best overall response of stable disease was reported in 5 patients (*n* = 3, 150 mg; *n* = 2, 200 mg), and the remaining 4 patients receiving 200 mg LY3023414 BID had progressive disease as best response.

Treatment duration for LY3023414 in patients with advanced disease and heavily pretreated tumor ranged from approximately 3 to 36 weeks. One patient with transitional cell carcinoma at the 200-mg BID dose level was on treatment for approximately 36 weeks. At baseline, this patient had Stage IV disease with aberrant histopathology and was previously treated with chemotherapies such as doxorubicin, gemcitabine, and paclitaxel.

## Discussion

This is the first study to evaluate tolerability and safety of LY3023414 in Japanese patients. In line with the global recommended dose of LY3023414 monotherapy, LY3023414 at 200 mg BID was safe and tolerable in Japanese patients with advanced malignancies. The overall AE profile observed in this study was consistent with the previous Phase 1 study, [12] and the majority of treatment-related AEs were mild or moderate in intensity. Grade ≥ 3 treatment-related AEs were not observed more than once in patients treated up to the maximum tolerated dose, supporting the tolerability of LY3023414 at the recommended Phase 2 dose level. The DLTs of stomatitis (Grade 3, non-serious) were observed in 2 patients on 200 mg BID; 1 patient recovered during follow-up on Study Day 36 and another patient withdrew from the study. Resumption of the study drug was permitted at a dose reduced by one level for patients who recovered from AEs within 2 weeks of study drug discontinuation. Stomatitis, mucositis, and mouth sores have been reported in most of the clinical studies with mTOR inhibitors (temsirolimus [75%], deforolimus [78%], and everolimus [41%]) [19]. In previous clinical studies, stomatitis (when not resulting in DLTs) has been manageable with supportive care, temporary omission, and dose reduction and has resolved despite treatment continuation [19]. The same management approach can be applied for LY3023414-induced stomatitis.

The frequency of stomatitis was 33.3% with the 200-mg dose of LY3023414 in the global study, less than that reported in this study with Japanese patients. The reason for this difference is unclear. The most commonly reported AEs of nausea, decreased appetite, and other gastrointestinal toxicities reported in this study are in line with clinical investigations of other PI3K/mTOR inhibitors [20–22].

One patient in this study experienced a serious AE of drug-induced ILD; this event was reversible by discontinuation of the study drug and treatment with intravenous corticosteroid. None of the patients experienced ILD in the global study. Mizuno and colleagues reported a high incidence of everolimus-induced ILD in Japanese patients with metastatic renal cell carcinoma [23]. An interim report on surveillance of everolimus-induced ILD in Japan reported an incidence of ILD of 17.4%. Dose-effect is one of the mechanisms proposed for the development of ILD; management of ILD includes mTOR inhibitor interruption or dose reduction and treatment with corticosteroid [24].

In this study, patients also experienced rash (33.3% in LY3023414 150-mg and 22.2% in LY3023414 200-mg arm) and hyperglycemia (66.7% in 150-mg and 22.2% in 200-mg arm). These AEs were manageable and consistent with the safety profile of other PI3K/mTOR inhibitors [25]. The overall safety results show no apparent dose relationship with the safety profile of LY3023414, and there were no new or unexpected safety findings in this study with Japanese patients.

Although no CR or PR were observed in this study, the disease control rate was reported in nearly half of the patients (55.6%, 5 of 9 patients) with different types of target lesions including lung (lung adenocarcinoma), hepatic lymph node (gallbladder adenocarcinoma), retroperitoneum (dedifferentiated liposarcoma), thymus gland (squamous cell carcinoma), and para-aortic lymph node tumors (transitional cell carcinoma). One patient with transitional cell carcinoma was on study treatment for approximately 36 weeks (253 days). The disease control rate of this study is similar to the global Phase 1 study (34.0%). Activated aberrant PI3K/AKT/mTOR signaling pathway results in tumor growth and survival. Monotherapy such as LY3023414 targets PI3K and mTOR simultaneously and inhibits the compensatory pathway, which obstructs tumor growth and survival [6, 7, 10].

The PK profile of LY3023414 was characterized by rapid absorption and elimination and was generally consistent with PK data reported in the global Phase 1 study [12], although the mean area under the drug plasma concentration versus time curve (AUC) values reported in this study are slightly higher than that reported in the global Phase 1 study [12]. This finding is likely explained by the fact that Japanese patients compared with patients from the global Phase 1 study had a lower average body surface area. Investigation of covariate relationships in the preliminary PK model built indicated that LY3023414 clearance was lower in patients with lower body surface area. The mean of PK exposure increased over time up to 4 h for both 150- and 200-mg doses. This pattern of increase over time was similar to that of mean changes in the PD markers. The increases in fasting glucose and C-peptide levels after LY3023414 administration was consistent with findings in the Phase 1 study. The PI3K/AKT/mTOR signaling pathway is a main regulator of enzymes involved in glucose, glutamine, and lipid metabolism [26, 27]. In context with hyperglycemia and hyperlipidemia, the rapamycin pathway is associated with insulin signaling, and mTOR inhibitors are likely to cause insulin resistance and prevent lipid clearance from blood, which results in high lipid and glucose levels. Metabolic events are common with mTOR inhibitors but manageable with supportive care [28]. Consequently, inhibition of the PI3K signaling pathway affects levels of these enzymes [29]. The validity of PD markers in these signaling is worth investigating in detail in further Phase 2 studies.

In conclusion, based on the results of this Phase 1 study in Japanese patients with advanced malignancies, 200 mg LY3023414 BID is the recommended Phase 2 dose in Japanese patients and warrants further clinical trials. LY3023414 monotherapy is currently under investigation for recurrent or persistent endometrial cancer with tumors harboring a known PI3K pathway activating mutation (NCT02549989).

## Abbreviations

AE: Adverse events
AUC: Area under the drug plasma concentration versus time curve
AUC_0-∞_: AUC time curve from time zero to infinity
AUC0-t_last_: AUC time curve from time zero to time t_last_, where t_last_ is the last time point with a measurable concentration
BID: Twice daily
CL/F: Apparent total body clearance
CL_ss_/F: Apparent total body clearance at steady state
C_max_: Maximum observed blood concentration
CR: Complete response;
DLT: Dose-limiting toxicity
ECOG PS: Eastern Cooperative Oncology Group performance status
ILD: Interstitial lung disease
mTOR: Mammalian target of rapamycin
PI3K: Phosphatidylinositol 3-kinase
PD: Pharmacodynamic
PK: Pharmacokinetic
PR: Partial response
t_1/2_: Half-life associated with the terminal rate constant (lambda z) in non-compartmental analysis
t_max_: Time of maximum observed blood concentration
V_z_/F: Apparent volume of distribution during the terminal phase
